# The role of cytochrome P450 gene rs1126742 polymorphism and risk of hypertension: a systematic review and meta-analysis

**DOI:** 10.1042/BSR20192513

**Published:** 2020-05-22

**Authors:** Ziyin Huang, Yufeng Jiang, Yafeng Zhou

**Affiliations:** Department of Cardiology, the First Affiliated Hospital of Soochow University, Suzhou City, Jiangsu Province, P. R. China

**Keywords:** CYP4A11, hypertension, meta-analysis, polymorphism, rs1126742, T8590C

## Abstract

**Background:** CYP4A11 gene T8590C (rs1126742) is proved to be an important locus that is relevant to hypertension. Various research on the relationship between rs1126742 polymorphism and hypertension have been published, but due to small sample sizes and limitations of the research objects, the combined results remain controversial.

**Methods:** We searched PubMed, Embase, OVID, Web of Science, Wan Fang, and CNKI databases for related articles. Three authors individually extracted data and the quality of studies was evaluated by using the 9-point Newcastle–Ottawa Scale (NOS) independently. Odds ratios (ORs) and 95% confidence intervals (CIs) were calculated in different genetic models by using a random-effect model or fixed-effect model according to inter-study heterogeneity. Besides, subgroup analysis and sensitivity analysis were performed and the publication bias was assessed.

**Results:** There were totally 12 independent case–control studies of 8673 cases and 6611 controls included. Significant associations were found between CYP4A11 gene T8590C polymorphism and hypertension under all genetic models (allele, homozygote, heterozygote, recessive, and dominant model). We also found that there was no obvious relationship between the rs1126742 polymorphism and hypertension in Asian. But positive association has been found in Caucasian in allele, homozygote, and recessive model.

**Conclusions:** CYP4A11 gene T8590C (rs1126742) polymorphism increases the occurrence of hypertension, particularly in Caucasian.

## Introduction

Hypertension (HTN) is a key risk factor for many common diseases, including stroke, renal failure, heart failure, and coronary heart disease [1]. And it has become one of the main sources of the global health burden [2]. It is well known that hypertension is a complex disease affected by a variety of genetic and environmental factors acting together [3]. So far, Genome-wide Association Studies (GWAS) have identified many loci related to blood pressure that provides a new idea for the treatment of hypertension [4–6]. Although lots of loci have been found and applied in clinic, there are still many gene variants remained to be included.

Cytochrome P450 is a superfamily of cysteine isozymes and a major mediator for oxidative transformation of many endogenous and exogenous molecules. CYP4A11 is a major member of cytochrome P450 superfamily and is classified as cytochrome P450 family 4. CYP4A11 is mainly used as an enzyme to convert arachidonic acid (AA) into 20-hydroxytetradecanoic acid (20-HETE) and 20-HETE plays a pivotal role in the regulation of hypertension [7–11]. Given the special role of 20-HETE, CYP4A11 gene variants have attracted wide attention. Rs1126742(T8590C) is one of the single nucleotide polymorphisms (SNPs) of the CYP4A11. Many studies have discussed the relationship between the rs1126742 polymorphisms and hypertension, but the combined result still remain controversial [12–23].

Because allele frequencies are often different, meta-analysis can help to compare genetic associations in different studies. Accordingly, we conducted this meta-analysis to further validate the relationship between T8590C (rs1126749) polymorphism and the risk of hypertension.

## Methods

### Search strategy

We followed Preferred Reporting Items for Systematic Reviews and Meta-Analyses (PRISMA) to perform this meta-analysis [24]. Relevant data were gathered from the electronic databases below: PubMed, OVID, Embase, Web of Science, Wan Fang, and CNKI databases up to July 1, 2019. The search strategy was based on a combination of the following terms: cytochrome P450, CYP4A11, rs1126742, T8590C, 20-HETE, polymorphism, genotype, genetic variant, high blood pressure, and hypertension. In addition, a manual search of relevant literature is conducted to identify studies other than computerized search.

### Inclusion and exclusion criteria

We reviewed all retrieved publications and citations, then evaluated the quality of each study by using the 9-point Newcastle–Ottawa Scale (NOS) independently [25]. We have pre-established criteria to evaluate obtained studies. The including study must meet the following criteria: case–control studies; studies that evaluated the association of rs1126742 polymorphism and hypertension susceptibility; studies that provided genotype distributions; studies that provide sufficient data to calculate odds ratios (ORs) and 95% confidence intervals (CIs). The exclusion criteria were: studies that provided too limited data for extraction, reviews, meta-analyses, abstracts-only articles, unpublished studies, and data duplicated in other studies.

### Data extraction

Three authors individually extracted useful data of each study in this meta-analysis. Any potential conflicts were resolved by discussion. Extraction of study data includes: first author’s name, ethnicities, publication year, age, gender, systolic and diastolic blood pressure of patients and control individuals, source of controls, genotyping method, and genotypes distribution. We made attempts to contact the original authors for detailed information, if the data were missed in the publication.

### Statistical analysis

We conducted Hardy–Weinberg equilibrium (HWE) tests of each study in our analysis for evaluation of included populations. We study the association strength between rs1126742 polymorphism and hypertension susceptibility by combining ORs and 95% CIs in a fixed or random-effect model based on the quantification of the heterogeneity calculated. The range of *I*^2^ is between 0 and 100, representing the degree of heterogeneity between studies. When *I*^2^ > 50 indicating heterogeneity between studies, a set analysis should be performed by using the random-effect model (Der Simonian and Laird methods). Otherwise the fixed-effect model (Mantel–Haenszel method) should be adopted. Subgroup analyses were also performed according to ethnicity, study sample size, source of control, and genotyping methods. The overall and subgroup analyses were both performed in five genetic models: allele (C vs. T), homozygote model (CC vs. TT), heterozygote model (TC vs. TT), recessive model (CC vs. TC+TT), and dominant model (CC+TC vs. TT). We have also investigated publication bias by calculating Egger’s test and drawing Begg’s funnel plot. We thought it was statistically significant between rs1126742 polymorphism with hypertension if *P*<0.05 or less. All statistical tests were performed by using Stata version 15.0 (Stata Corporation).

## Results

### Study characteristics

**Figure 1:**
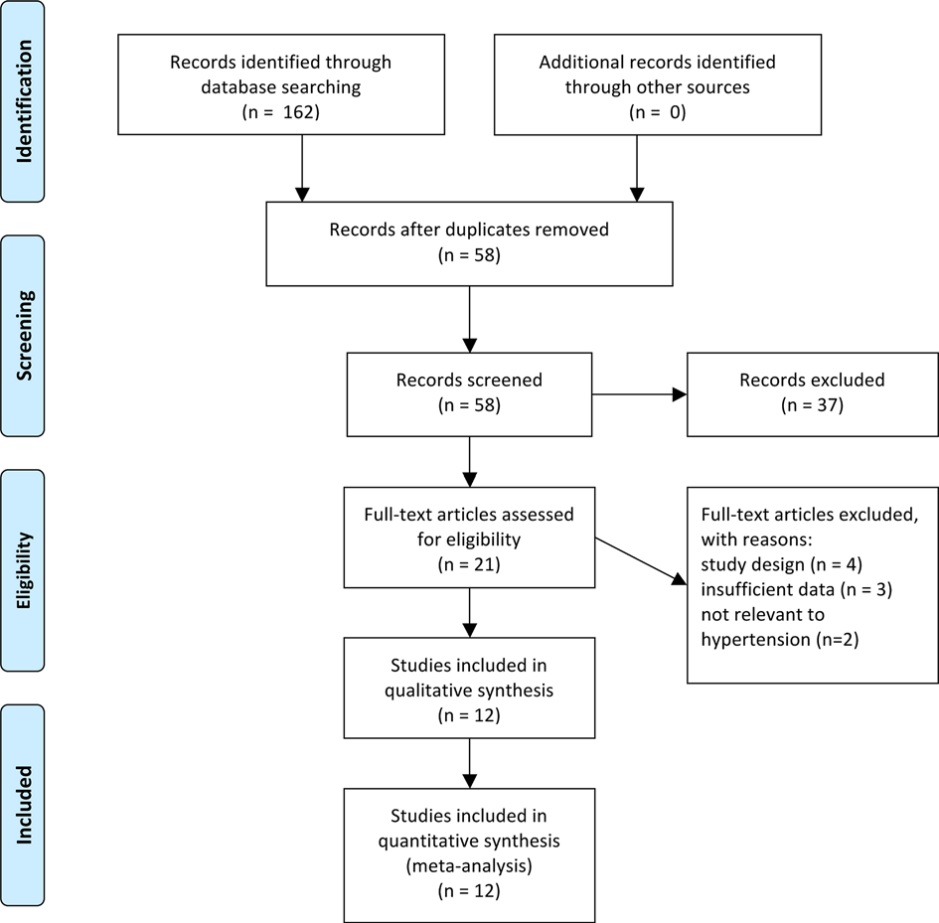
The PRISMA flow diagram of the study selection and exclusion

We search 6 databases and 162 records were found in total. After deleting duplicated studies, there were 58 studies remained for screening and 104 records were excluded. About 21 studies were read in full, and 9 of full-text articles were excluded because of unmatched study design (*n* = 4), insufficient data (*n* = 3), and not relevant to hypertension (*n* = 2). Figure 1 shows the complete process of the selection and exclusion. Finally, 12 studies of 8673 cases and 6611 controls were included in this meta-analysis. Table 1 shows the characteristics of studies in this meta-analysis. The sample sizes ranged from 235 to 5975 of all eligible studies. The results of NOS are shown in Table 1. In our meta-analysis, the NOS of all eligible studies was >6 points, representing a good study quality. Table 2 shows the genotype distribution and allele frequency in the case and control of each study.

**Table 1:** Characteristics of the studies included for meta-analysis

Author	Year	Ethnicity	Age (years)		Gender (M/F)		SBP (mmHg)		DBP (mmHg)		Source of control	Genotyping method	NOS score	HWE*
			Case	Control	Case	Control	Case	Control	Case	Control				
Gainer et al.	2005	Caucasian	M: 38.2 ± 7.2	M: 46.8 ± 13.2	30/30	29/31	Case: M: 108.9 ± 11.7; F: 110.2 ± 16.1^#^				PB	Direct sequencing	7	0.55
			F: 38.9 ± 7.3	F: 45.2 ± 12.4			Control: M: 90.2 ± 7.4; F: 86.1 ± 9.1^#^							
Mayer et al.	2005	Caucasian	M: 56.7 ± 12.8	M: 34.5 ± 8.7	367/282	346/402	M: 149.6 ± 17.3	M: 124.4 ± 9.6	M: 88.2 ± 12.2	M: 77.0 ± 7.6	PB	TaqMan	8	0.87
			F: 59.4 ± 10.0	F: 37.1 ± 8.8			F: 149.7 ± 16.2	F: 118.5 ± 11.0	F: 85.4 ± 11.6	F: 73.8 ± 8.0				
Mayer et al.	2006	Caucasian	57 ± 7	56 ± 8	202/26	287/45	147 ± 14	122 ± 10	93 ± 9	78 ± 7	HB	Direct sequencing	7	0.33
Fava et al.	2008	Caucasian	57.2 ± 5.9		2261/3293		140.4 ± 18.7		86.6 ± 9.3		HB	Endpoint fluorescent	8	0.62
Fu et al .	2008	Asian	59.1 ± 10.0	60.9 ± 11.9	211/93	136/71	164.6 ± 23.2	115.5 ±± 9.7	97.8 ± 16.6	70.2 ± 7.8	HB	TaqMan	7	0.53
Sugimoto et al.	2008	Asian	65.6 ± 8.0	65.7 ± 8.0	228/267	232/262	157.5 ± 16.0	123.2 ± 11.9	85.2 ± 11.0	71.1 ± 9.1	HB	Direct sequencing	8	0.66
Ward et al.	2008	Caucasian	58.2 ± 9.5	55.6 ± 8.6	97/64	32/42	137	116	80	71	HB	Direct sequencing	7	0.63
Fan et al.	2011	Asian	M: 58.4 ± 12.3	M: 51.7 ± 11.0	312/378	292/364	–	–	–	–	PB	TaqMan	8	0.65
			F: 61.6 ± 12.3	F: 49.9 ± 10.1										
Williams et al.	2011	Mixed	45.7 ± 10.5	44.7 ± 11.8	258/221		148.7 ± 20.8	110.5 ± 12.3	89.6 ± 12.3	66.2 ± 8.7	HB	Sequenom iPlex	8	0.52
Yan et al.	2013	Asian	62.7 ± 11.2	63.2 ± 8.4	188/140	146/151	137.4 ± 14.6	110.9 ± 10.0	78.2 ± 10.0	67.3 ± 7.2	HB	High resolution melting	7	0.86
Yang et al.	2014	Asian	60.9 ± 10.4	60.7 ± 9.2	523/341	422/239	161.3 ± 15.1	114.8 ± 9.7	95.9 ± 8.5	71.3 ± ± 7.4	PB	TaqMan	8	0.73
Zhang et al.	2017	Asian	56.8 ± 14.7	55.9 ± 15.1	382/438	406/422	–	–	–	–	HB	TaqMan	8	0.21

Year, publication year. Abbreviations: DBP, diastolic blood pressure; F, female; HB, hospital based; HWE, Hardy–Weinberg equilibrium; M, male; NOS, Newcastle–Ottawa scale; PB, population based; SBP, systolic blood pressure.

Data are presented as mean ± standard derivation.

Case–control design was used in all the included studies.

* *P* value for Hardy–Weinberg equilibrium test in controls.

# Mean arterial pressure.

**Table 2:** Genotype distribution and allele frequency of cytochrome P450 gene family polymorphisms in cases and controls

Author	Genotype (N)								Allele Frequency (*N*, %)					
	Cases				Controls				Cases			Controls		
CYP4A11 rs1126742	Total	TT	TC	CC	Total	TT	TC	CC	T	C	RAF	T	C	RAF
Gainer	195	126	64	5	197	152	41	4	316	74	19.0%	345	49	12.4%
Mayer	649	481	149	19	748	574	164	10	1111	187	14.4%	1312	184	12.3%
Mayer	228	152	68	8	332	250	79	3	372	84	18.4%	579	85	12.8%
Fava	3805	2924	800	81	2170	1665	475	30	6648	962	12.6%	3805	535	12.3%
Fu	304	179	122	3	207	133	64	10	480	128	21.1%	330	84	20.3%
Sugimoto	495	325	157	13	494	326	153	15	807	183	18.5%	805	183	18.5%
Ward	161	115	41	5	74	54	19	1	271	51	15.8%	127	21	14.2%
Fan	684	378	259	47	654	393	231	30	1015	353	25.8%	1017	291	22.2%
Williams	332	227	94	11	147	109	34	4	548	116	17.5%	252	42	14.3%
Yan	328	175	132	21	297	150	123	24	482	174	26.5%	423	171	28.8%
Yang	864	510	299	55	661	401	230	30	1319	409	23.7%	1032	290	21.9%
Zhang	628	325	242	61	630	413	188	29	892	364	29.0%	1014	246	19.5%

Case–control design was used in all the included studies; RAF, risk allele frequency.

### Association of CYP4A11 gene T8590C (rs1126742) gene polymorphism with hypertension

**Figure 2:**
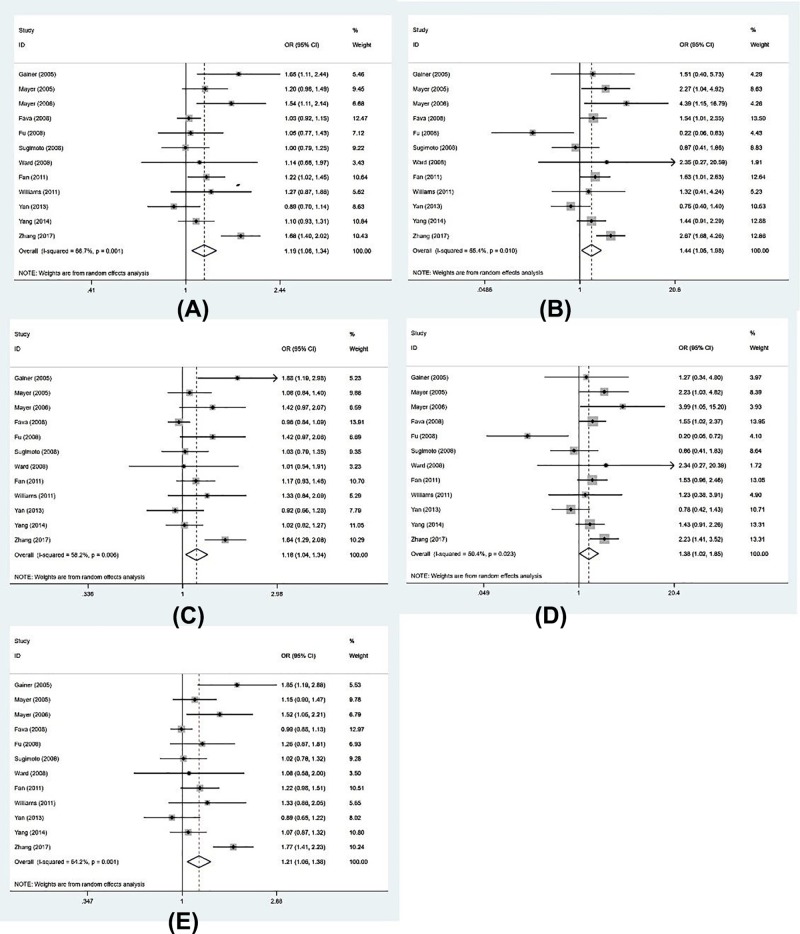
Forest plot on the association of rs1126742 polymorphism and hypertension risk Forest plot from the meta-analysis on the association of rs1126742 polymorphism and hypertension risk in (**A**) allele model: C vs. T; (**B**) homozygote model: CC vs. TT; (**C**) heterozygote model: TC vs. TT; (**D**) recessive model: CC vs. TC+TT; and (**E**) dominant model: TC+CC vs. TT; CI, confidence interval, OR, odds ratio.

We chose random effect model to conduct the data analysis when *I*^2^ > 50%. This meta-analysis indicated significant association between rs1126742 and hypertension under allelic (OR = 1.19, 95%CI = 1.06–1.34, *P* = 0.004, *I*^2^ = 66.7%), homozygote (OR = 1.44, 95%CI = 1.05–1.98, *P* = 0.024, *I*^2^ = 55.4%), heterozygote (OR = 1.18, 95%CI = 1.04–1.34, *P* = 0.012, *I*^2^ = 58.2%), recessive (OR = 1.38, 95%CI = 1.02–1.85, *P* = 0.035, *I*^2^ = 50.4%), and dominant genetic model (OR = 1.21, 95%CI = 1.06–1.38, *P* = 0.005, *I*^2^ = 64.2%). In summary, our meta-analysis proved that rs1126742 polymorphism of the CYP4A11 gene significantly increases the risk of hypertension. The results of forest plot are shown in Figure 2. We also conducted subgroup analyses according to ethnicity, source of control, sample size, and genotyping method. The detailed information was presented in Table 3. In subgroup analysis of ethnicity, an obvious association between re1126742 mutation and the risk of hypertension was discovered in Caucasian group in allelic, homozygote, and recessive models (allelic: OR = 1.24, 95%CI = 1.03–1.50, *P* = 0.026; homozygote: OR = 1.79, 95%CI = 1.27–2.52, *P* = 0.001; recessive: OR = 1.76, 95%CI = 1.26–2.48, *P* = 0.001) excluding heterozygote (OR = 1.18, 95%CI = 0.94–1.48, *P* = 0.159), and dominant genetic model (OR = 1.23, 95%CI = 0.99–1.54, P = 0.068). But there was no obvious relationship between the rs1126742 polymorphism and hypertension in Asian (allelic: OR = 1.14, 95%CI = 0.95–1.37, *P* = 0.147; homozygote: OR = 1.16, 95%CI = 0.70–1.92, *P* = 0.555; heterozygote: OR = 1.17, 95%CI = 0.98–1.40, *P* = 0.075; recessive: OR = 1.12, 95%CI = 0.71–1.78, *P* = 0.626; dominant genetic model: OR = 1.18, 95%CI = 0.97–1.44, *P* = 0.093). In subgroup analysis of source of control, we found population-based controls showed significant increased risk of hypertension in some models (allelic: OR = 1.20, 95%CI = 1.08–1.33, *P* = 0.001; homozygote: OR = 1.62, 95% CI = 1.20–2.18, *P* = 0.001; heterozygote: OR = 1.14, 95%CI = 1.00–1.29, *P* = 0.05; recessive: OR = 1.56, 95%CI = 1.16–2.09, *P* = 0.003; dominant genetic model: OR = 1.19, 95%CI = 1.05–1.34, *P* = 0.01).

**Table 3:** Subgroup analyses of association between cytochrome P450 gene family polymorphisms and hypertension

Subgroup		CYP4A11 rs1126742			
		Odds ratio	95% Confidential interval	*P* value	*I*^2^ (%)
**Allele model**					
Source of control	HB	1.17	(0.98, 1.40)	0.09	75.8
	PB	1.20	(1.08, 1.33)	0.001	13.3
Sample size	≥500	1.16	(1.02, 1.32)	0.02	72.9
	<500	1.38	(1.08, 1.76)	0.01	0.0
Genotyping method	PCR	1.22	(1.04, 1.43)	0.02	58.7
	TaqMan	1.25	(1.05, 1.47)	0.01	70.1
**Homozygote model**					
Source of control	HB	1.29	(0.76, 2.17)	0.34	70.1
	PB	1.62	(1.20, 2.18)	0.001	0.0
Sample size	≥500	1.42	(0.98, 2.05)	0.06	67.3
	<500	1.52	(0.68, 3.43)	0.31	0.0
Genotyping method	PCR	1.43	(0.82, 2.48)	0.21	33.9
	TaqMan	1.55	(0.93, 2.57)	0.09	71.2
**Heterozygote model**					
Source of control	HB	1.18	(0.98, 1.42)	0.07	65.7
	PB	1.14	(1.00, 1.29)	0.05	48.1
Sample size	≥500	1.14	(1.00, 1.30)	0.05	60.7
	<500	1.44	(1.08, 1.92)	0.01	23.9
Genotyping method	PCR	1.23	(1.02, 1.49)	0.03	48.9
	TaqMan	1.23	(1.03, 1.47)	0.02	59.7
**Recessive model**					
Source of control	HB	1.22	(0.75, 1.99)	0.42	66.3
	PB	1.56	(1.16, 2.09)	0.003	0.0
Sample size	≥500	1.37	(0.97, 1.93)	0.08	63.4
	<500	1.38	(0.62, 3.09)	0.43	0.0
Genotyping method	PCR	1.35	(0.78, 2.34)	0.28	27.6
	TaqMan	1.45	(0.89, 2.35)	0.13	69.2
**Dominant model**					
Source of control	HB	1.20	(0.99, 1.46)	0.07	72.5
	PB	1.19	(1.05, 1.34)	0.01	38.9
Sample size	≥500	1.17	(1.02, 1.35)	0.03	68.8
	<500	1.45	(1.10, 1.91)	0.01	8.8
Genotyping method	PCR	1.25	(1.04, 1.50)	0.02	55.4
	TaqMan	1.27	(1.06, 1.53)	0.01	65.9

Abbreviations: HB, hospital based; PB, population based; PCR, polymerase chain reaction.

### Sensitivity analysis

**Figure 3:**
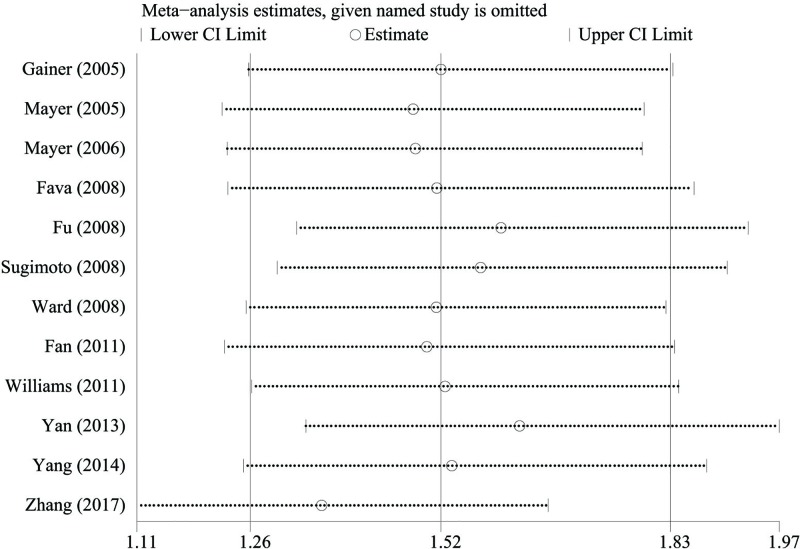
Sensitivity analysis of the pooled OR coefficients on the relationship between rs1126742 polymorphism and hypertension risk Abbreviations: CI, confidence interval; OR, odds ratio.

We performed sensitivity analysis to evaluate the stability of the results in the association between rs1126742 and hypertension susceptibility by removing one study at each round of the analysis. As it is shown in Figure 3, no altered results are showed after the individual study omitted. This suggests that this meta-analysis provides more reliable evidence to prove the association between the rs1126742 and hypertension.

### Publication bias

**Figure 4:**
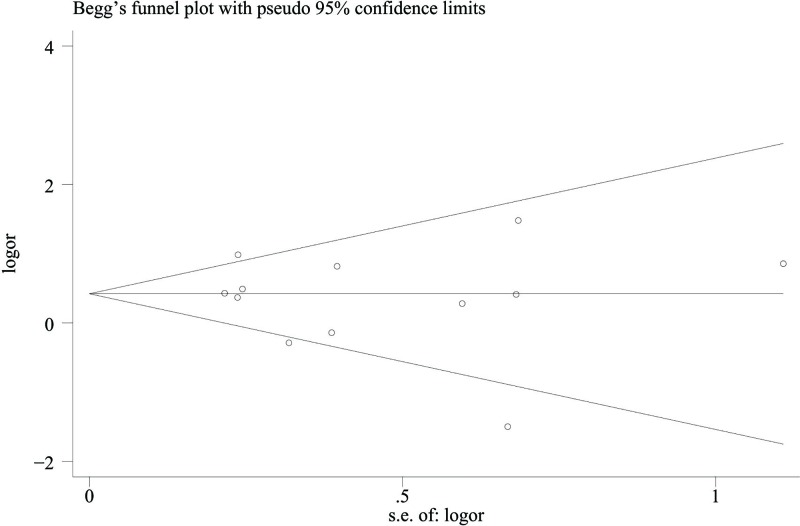
Begg’s plot with pseudo 95% confidence limits in recessive model

Publication bias is a common problem in meta-analysis. In our meta-analysis, we calculated Egger’s test and drew the Begg’s funnel plot to evaluate the publication bias. Visually from the Begg’s funnel plot (Figure 4), we could see all the 12 studies were symmetrically distributed on the two sides, which indicated no publication bias in our meta-analysis. Meanwhile, Egger’s test showed the same results (all *P*>0.05).

## Discussion

CYP4A11 is one of the main enzymes that synthesizes 30% of 20-HETE in human body. The transfer to cytosine in nucleotide 8590 of exon 11 results in a nonsynonymous phenylalanine (F) to serine (S) substitution at residue 434 of CYP4A11. The change of protein 434 F to S affects the oxidation of arachidonic acid to 20-HETE [26]. 20-HETE may act as a strong vasoconstrictor in the renal arterioles and it may function as a natriuretic, antihypertensive substance in renal tubules. We may conclude that 20-HETE affected the formation of hypertension and single-nucleotide polymorphisms (SNPs) of CYP4A11 can explain susceptibility of hypertension.

Many studies have tried to discover a possible association between the rs1126742 polymorphism and hypertension. Mayer et al. [13] revealed that C allele variant in CYP4A11 was a risk factor for hypertension. Fava et al. [15] also reported that CC genotype had a higher risk of hypertension in Swedish subjects, while other studies suggested the contrary result. At the same time, one study conducted by Ward et al. [18] found that there was no obvious relationship between the CYP4A11 T8590C polymorphism and the risk of hypertension in Australians. As a result of the situation described above, we collected 12 studies to conduct this meta-analysis in order to provide a combined result. The result reveals that CYP4A11 T8590C polymorphism can increase the risk of hypertension in all models. We also conducted subgroup analyses according to ethnicity, source of control, sample size, and genotyping method. We found that there was no obvious relationship between the rs1126742 polymorphism and hypertension in Asian. But positive association has been found in Caucasian in allele, homozygote, and recessive model. Relatively large heterogeneity was present in this meta-analysis, perhaps due to the limited study sample sizes. The heterogeneity was decreased in ethnicity subgroup analysis. It is suggested that the ethnicity may be the source of heterogeneity. According to previously published animal studies, clear evidence exists that sex hormones, especially testosterone, modulate CYP4A11 expression and activity contributing to higher blood pressure levels. In the other word, enzymes involved in 20-HETE biosynthesis and metabolism may differ between males and females. And CYP4A11 gene rs1126742 variants may also have a role in hypertension with a gender-specific effect. However, few genetic studies include gender as a variable in data analysis and previous studies referring to this issue did not get a definite conclusion due to the small sample sizes. Because of the lack of individual patient data, the subgroup meta-analysis of gender couldn’t be carried out in the present study. Large scale studies are needed to further clarify whether the CYP4A11 T8590C polymorphism has a gender-specific effect.

Understanding the genetic background of CYP4A11 is of great significance for better clinical application in the near future. People with high genetic risk scores may increase occurrence of hypertension. So we can use this genetic locus to predict hypertension. Second, some people believe that rs1126742 might be a promising locus for genetic therapy of hypertension [27].

There are some limitations we can’t ignore. First, the data were not further stratified in subgroup by alcohol consumption, age, gender, smoking and other lifestyle factors, because the included studies in this research lacked consistent baseline data. Second, hypertension is a complex disease involved in several genes as well as many interferential factors such as environmental factors, which could influence the final results. Third, all the studies included were conducted in Asian and Caucasian, so people in other ethnicity did not attend this meta-analysis which may make our study localized. Taken these limitations into consideration, the results we concluded should also be interpreted with caution.

## Conclusions

The current meta-analysis concluded that the rs1126742 polymorphism of CYP4A11 significantly increases the risk of hypertension, particularly in Caucasian. It could be a promising locus for clinical genetic diagnosis and therapy of hypertension in the future. But more case–control studies with high quality are needed to strengthen the findings of this meta-analysis.

## Abbreviations

20-HETE: 20-hydroxytetradecanoic acid
AA: arachidonic acid
CI: confidence interval
HWE: Hardy–Weinberg equilibrium
NOS: Newcastle–Ottawa Scale
OR: odds ratio
SNP: single nucleotide polymorphism
